# Machine Intelligence in Single-Cell Data Analysis: Advances and New Challenges

**DOI:** 10.3389/fgene.2021.655536

**Published:** 2021-05-31

**Authors:** Jiajia Liu, Zhiwei Fan, Weiling Zhao, Xiaobo Zhou

**Affiliations:** ^1^College of Electronic and Information Engineering, Tongji University, Shanghai, China; ^2^School of Biomedical Informatics, The University of Texas Health Science Centre at Houston, Houston, TX, United States; ^3^West China School of Public Health, West China Fourth Hospital, Sichuan University, Chengdu, China

**Keywords:** data imputation, batch effects removal, cell cycle identification, cell type identification, CNV estimation, trajectory inference, cell–cell interaction, regulatory network inference

## Abstract

The rapid development of single-cell technologies allows for dissecting cellular heterogeneity at different omics layers with an unprecedented resolution. In-dep analysis of cellular heterogeneity will boost our understanding of complex biological systems or processes, including cancer, immune system and chronic diseases, thereby providing valuable insights for clinical and translational research. In this review, we will focus on the application of machine learning methods in single-cell multi-omics data analysis. We will start with the pre-processing of single-cell RNA sequencing (scRNA-seq) data, including data imputation, cross-platform batch effect removal, and cell cycle and cell-type identification. Next, we will introduce advanced data analysis tools and methods used for copy number variance estimate, single-cell pseudo-time trajectory analysis, phylogenetic tree inference, cell–cell interaction, regulatory network inference, and integrated analysis of scRNA-seq and spatial transcriptome data. Finally, we will present the latest analyzing challenges, such as multi-omics integration and integrated analysis of scRNA-seq data.

## Introduction

**FIGURE 1:**
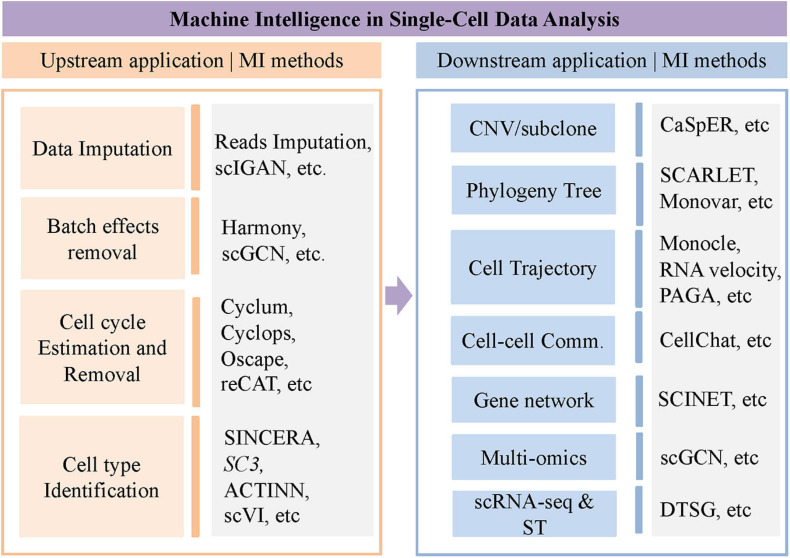
Brief summary of computational methods for single cell data analysis.

The rapid development of single-cell sequencing technologies makes it possible to explore cell heterogeneity of genome, epigenome, and transcriptome, and cell–cell interaction/communication in the context of a specific environment in a tissue. Due to various technical noise such as dropout rate, it is pretty challenging to measure the expression level in a single cell accurately. Therefore, model-based imputation methods are needed for data imputation to clean the technical noise and correct false expression and dropout events. In addition, most of the downstream analyses of the single-cell sequencing data, such as the reconstruction of differentiation trajectory, analysis of cell–cell interaction, etc., require computational tools and models. In this review, we will summarize the latest progress of single-cell sequencing data analysis from a machine learning viewpoint, including scRNA-seq data imputation, batch effect removal, cell cycle and cell type identification, copy number estimate, trajectory inference, cell–cell interaction, and regulatory network inference. These applications are briefly summarized in Figure 1.

## Single-Cell Sequencing Data Imputation and Compositional Data Analysis

### Accurate Quantification of Transcript for scRNA-seq With Reads Imputation

**FIGURE 2:**
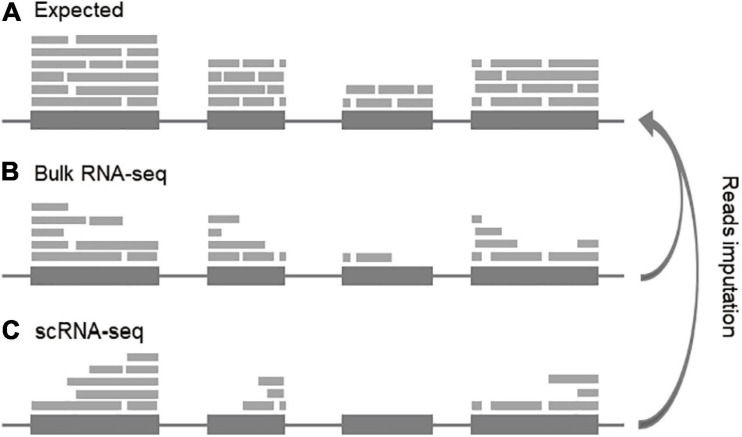
Conceptual view of reads imputation. **(A)** Expected distribution of transcripts. **(B)** Reads notches in bulk RNA-seq. **(C)** Reads notches in scRNA-seq. For concise, the junction reads are not indicated.

As a new generation of high-throughput sequencing technology, RNA sequencing makes it possible to provide insight into the transcriptome of a population of cells or a single cell (Xu and Zhou, 2018). RNA-seq technology can generate short reads from a set of fragments of transcribed molecules in a sample. The basic assumption of RNA-seq for transcript quantitation is that the number of RNA molecules in a given transcript can be proportionally represented as reads generated from randomly sampled fragments, either from the single end or paired ends. Therefore, RNA-seq is basically a sub-sampling process, through which it is expected that the reads will compatibly distribute along with the transcripts and the counts will represent the true expression distribution of all transcripts in a given sample (Figure 2A). However, each step of RNA-seq introduces bias (uneven reads distribution), leading to exons not evenly covered by reads. We call these reads notches (Figures 2B,C). Obtaining accurate gene expression requires filling up the read notches along the gene to make sure the compatible coverages within and among exons and transcripts (Figure 2A), especially, for reads-based metrics (such as RPKM, FPKM, and also TPM). Since these metrics are just arithmetically average the reads of the entire transcript, if the read notches are not filled, the expression level will be underestimated. Recovery of the missing reads from these data will largely enhance the detection and quantification power of scRNA-seq data.

ReadsImpute (Xu, 2018) is the first tool that imputes the missing reads and gives a more accurate transcript and gene quantifications. It implements capacity expansion on the residual flow network derived from the standard max-flow optimization on the initial flow network, and maximizes transcript quantification by imputing missing reads. Comparing with many popular approaches, such as Stringtie (Pertea et al., 2015), Salmon (Patro et al., 2017), and Kallisto (Bray et al., 2016), ReadsImpute appears to be a better method in achieving consistent quantifications between the subsamples and entire samples after reads imputation. StringTie applies a traditional network flow algorithm to assemble complex datasets into transcripts (Pertea et al., 2015). Salmon combines a new dual-phase parallel inference algorithm and feature-rich bias models for quantifying transcript abundance from RNA-seq reads (Patro et al., 2017). Salmon is an ultra-fast method since it takes advantage of multiple CPU cores. Kallisto is designed based on pseudo alignment to assemble transcripts both from bulk and single-cell RNA-Seq data (Bray et al., 2016). More reads imputation methods are needed to be developed.

### Recover Dropout Events in Single-Cell Transcriptome Profiles

“Dropout” is another most important imperfect that hinders the power of scRNA-seq, where a lot of low-abundant information will be lost at expression level (Kharchenko et al., 2014). Usually, these dropouts occur due to a variety of reasons, for example, RNA cannot be reversely transcribed into DNA or PCR amplification of cDNA is failed during the scRNA-seq process, causing a truly expressed transcript cannot be detected during sequencing. Thus, it is necessary to correct the false zero or missing expression due to dropout events in scRNA-seq data using computational imputation methods.

Many methods and tools are currently available for solving the dropout issues of scRNA-seq data (Pierson and Yau, 2015; Azizi et al., 2017; Lin et al., 2017; Chen and Zhou, 2018; Gong et al., 2018; Huang et al., 2018; Li and Li, 2018; Ronen and Akalin, 2018; van Dijk et al., 2018; Amodio et al., 2019; Arisdakessian et al., 2019; Eraslan et al., 2019; Gunady et al., 2019; Peng et al., 2019; Tracy et al., 2019; Wagner et al., 2019; Badsha et al., 2020; Marouf et al., 2020; Xu et al., 2020). MAGIC recovers dropout events by using diffusion geometry to share similarities across cells (van Dijk et al., 2018). VIPER borrows information from a sparse set of local neighborhood cells of similar expression patterns to impute the expression measurements in the cells of interest based on non-negative sparse regression models (Chen and Zhou, 2018). DeepImpute and SAUCIE (Amodio et al., 2019; Arisdakessian et al., 2019) were developed for scRNA-seq data imputation with deep learning methods. These approaches adopted node/gene interaction structure, which could be seriously overfitted due to the limited single cell numbers. Generative Adversarial Network (GAN), widely used in the field of image processing, is also a powerful tool for single-cell analysis (Bonn et al., 2018; DePasquale et al., 2018; Ghahramani et al., 2018; Marouf et al., 2020). Marouf et al. designed a conditional single-cell generative adversarial neural network (cscGAN) to generate realistic single-cell RNA-seq data based on fully connected neural networks. Gene-to-gene associations from cell types are captured to generate specific types of cells. WGANs were applied to optimize object function. However, the fully connected network in cscGAN can not guarantee the performance in data imputation for specific dropouts. Ghahramani et al. applied GAN to simulate scRNA-seq data to cover the diversity of different cell types (Pierson and Yau, 2015; Lin et al., 2017; Huang et al., 2018; Li and Li, 2018; Ronen and Akalin, 2018; Badsha et al., 2020; Xu et al., 2020). We developed a novel GANs approach, named scIGANs for scRNA-seq imputation (Xu, 2018). Unlike common imputation algorithms, scIGANs uses generated cells rather than observed cells to maintain a balance between major and rare cell populations. scIGANs learns the distribution of gene expression data under a conditional GANs framework and imputes the dropout events from the expression matrix. Using either simulated or real scRNA-seq data, our analysis indicated that scIGANs significantly enhanced various downstream analyses compared to existing imputation algorithms.

### Compositional Analysis of Single-Cell RNA-seq Data

So far, compositional analysis has been an active and ongoing area in metagenomic data (Norouzi-Beirami et al., 2021) and microbiome research (Chen and Li, 2013; Bian et al., 2017; Rivera-Pinto et al., 2018), due to the compositional nature of metagenomic and microbiome data. This is also opens a new perspective on the analysis of single cell RNA-seq data. RNA-seq data are compositional in nature since the abundances for each sample are limited by the library size (Wu et al., 2021), this is also true for single cell RNA-seq data. The estimated transcript abundance relies on several factors and the count data are not actually counts, but rather components of a whole in scRNA-seq data (Quinn et al., 2018). scRNA-seq data can be regarded as compositional data, which measure each sample as a composition with non-zero positive values carrying relative information (Aitchison, 1982). Thus, an additional normalization step that corrects for the arbitrary library sizes need to be taken into account when analysis compositional counts of scRNA-seq data. Several effective normalization methods have been applied to single cell compositional data analysis, such as normalization to effective library size, log-ratio transformation and “normalization,” etc., for compositional data (Quinn et al., 2018).

## Batch Effects Removal and Data Integration for Single-Cell Multi-Omics

Datasets produced in different laboratories at different times and handled by different protocols and technologies contain batch processing effects, affecting data integration and interpretation, and deriving misleading outcomes. Therefore, removing batch effects is a critical step before conducting further data analyses. Here, we will introduce some batch effects removal and data integration algorithms for multi-omics and cross-platform single-cell sequencing data.

### Computational Methods for Single-Cell Multi-Omics Integration

Different omics platforms have different types of attributes and distributions, which makes it challenging to integrate them. They can be continuous variables such as RNA-seq, microRNA-seq, and ATAC-seq, binary variables such as SNPs, discretized variables such as CNVs, graphs such as pathway networks and metabolic pathways, and characters such as binding sites. Individual omics data can only provide limited insights into the biological mechanisms of disease. A comprehensive understanding of the key mechanisms underlying biological processes relies on an integrative analysis from multi-omics data.

Many machine learning methods are developed so far to integrate multi-omics data, such as Bayesian approach, heterogeneous graph approaches such as matrix factorization-based algorithms, deep learning approaches, and other machine learning approaches. LIGER (Welch et al., 2019), an algorithm for delineating shared and dataset-specific features of cell identity, was developed to integrate scRNA-seq and DNA methylation profiles to reveal the putative mechanisms of cell-type-specific epigenomic regulation within their defined mouse cortical cell types. Nativio et al. (2020) identified molecular pathways and epigenetic alterations underlying late-onset Alzheimer’s disease by integrating transcriptomic, proteomic, and epigenomic profiling of human brains. Bian et al. (2018) reconstructed genetic lineages and traced the epigenomic and transcriptomic dynamics through single-cell multi-omics. Granja et al. (2019) identified both patient-shared malignant signatures and patient-specific regulatory features such as RUNX1-linked regulatory elements via integrating single-cell transcriptomic and chromatin-accessibility profiles in acute leukemia analysis. Transfer learning is a field of machine learning and is currently widely used for batch effect removal of single-cell data. Wang C. et al. (2020) developed a Model-based Analyses of Transcriptome and Regulome (MAESTRO) for integrative analyses of scRNA-seq and scATAC-seq data from multiple platforms. MAESTRO aims at integration based on cell clusters of datasets from different platforms. Song et al. (2020) applied semi-supervised graph convolutional networks, termed single-cell Graph Convolutional Network (scGCN), to implement transfer learning. scGCN captures topological cell relations to learn the order and topological structure of cells in high resolution to improve integration performance. scGCN can reliably integrate single-cell datasets and transferring labels across studies by considering knowledge graphs. Thus, the information learned from previous datasets can be transferred into the new datasets.

Other algorithms such as Autoencoder can also implement transfer learning well. Li et al. (2020) couple a deep autoencoder with a soft cluster algorithm to embed scRNA-seq data by gradually removing batch effects. Recently, Batch Effect Removal Using Deep Autoencoders (BERMUDA) was proposed for batch effect correction of scRNA-seq data (Wang T. et al., 2019). BERMUDA treats scRNA-seq data from different batches as different domains and uses domain adaptation methods in transfer learning to reduce different scRNA-seq datasets to the same low-dimensional space and then remove batch effects in the low dimension.

### Integration of Cross-Platform Single-Cell Sequencing Data

As the first wave of single-cell multi-omics technology, scRNA-seq allows the transcriptomic measurement in thousands of single cells from different biological samples under varieties of sequencing technologies and platforms (Muraro et al., 2016; Azizi et al., 2018; Buenrostro et al., 2018; Cusanovich et al., 2018; Tabula Muris Consortium et al., 2018). Multiple single-cell sequencing data have been obtained in recent years, such as scRNA-seq, single-cell DNA sequencing (scDNA-seq), single-cell DNA methylation sequencing (scMethylation-seq), and single-cell transposase-accessible chromatin (scATAC-Seq). For scRNA-seq, different protocols have been developed to quantify single-cell transcriptomes, such as Smart-seq2 and 10X Chromium, Drop-seq, etc. Since the general batch effect removal algorithms may not be simply applied to single-cell sequencing data, some computational methods have been developed to address the challenges of cross-platform/protocol single-cell sequencing data integration (Butler et al., 2018; Kiselev et al., 2018; Barkas et al., 2019; de Kanter et al., 2019; Stuart et al., 2019; Song et al., 2020). These methods extract shared information from individual cells across different datasets, but ignore the differences between datasets. Tools developed for the batch correction of microarray data such as ComBat and Limma have been applied to eliminate the influences of batches on scRNA-seq data (Jaakkola et al., 2017; Risso et al., 2018). Limma package contains strong tools for reading and normalizing data and can be applied to several kinds of analysis of scRNA-seq data, such as differential expression and differential splicing analysis (Ritchie et al., 2015). SAVER-X, combined a deep autoencoder with a Bayesian model, extracts transferable gene–gene relationships across datasets generated from different laboratories. The trained network can be applied to new data, thereby improving data quality (Wang J. et al., 2019). Tran et al. (2020) compared 14 batch-effect correction methods based on time-consuming computing ability, large datasets handling ability, and batch-effect correction accuracy for scRNA-seq data generated by different sequencing technologies, such as smart-seq2, inDrop, 10X, and CEL-seq, etc. They found that Harmony (Korsunsky et al., 2019), LIGER (Welch et al., 2019), and Seurat (Butler et al., 2018; Stuart et al., 2019) had better performance for batch integration, and Harmony was recommended as the first method to apply considering its significantly shorter runtime. Harmony adopts a simple clustering strategy by iteratively removing batch effects. The cells with similar expression signatures but from different batches were clustered together while trying to maximize batch diversity within each cluster, and then the dataset correction factor is calculated during each iteration. Harmony also performs well on data integration in terms of short runtime and less memory consumption. The only drawback is that it cannot integrate datasets with different number of cells. However, LIGER, Seurat, and harmony can only handle current data, but the trained model/parameters cannot be applied to new data. The methods used transfer learning can solve this problem, such as SAVER-X, BERMUDA, and scGCN, etc. The network/model trained on the current data can be used for new data, thereby diluting the differences between datasets. The performances of the above methods are all evaluated on different datasets generated by different protocols/platforms.

## Cell Cycle Identification

The cell cycle is a key component in the biological processes, which drivers the transcriptional heterogeneity in cell differentiation (Pauklin and Vallier, 2013), cell state and oncogenesis (Kastan and Bartek, 2004; Bar-Joseph et al., 2008). Studying the assignment of cell cycle phases is also of great significance to the occurrence, development and treatment of tumors. Consequently, accurately identifying the cell cycle phases is the key to understand different biological processes (Scialdone et al., 2015).

At present, most researches use cell cycle information obtained from experiments, such as utilizing chemical induction (Vassilev, 2006), counterflow centrifugation elutriation (Ly et al., 2014), and DNA content (Sasagawa et al., 2013) to investigate the enrichment of cells in different cell cycle phases. However, these methods have the disadvantages of complicated operation, low sensitivity, long experiment period, and may introduce biological batch effects. Therefore, computational tools have been developed to allocate cells to their cell cycle stages based on their transcriptional profiles.

### Computational Methods to Predict Cell Cycle Phases

Several machine learning methods have been developed to analyze cyclic or circadian processes on the single-cell resolution, including continuous and discrete predictions of cell cycle phases. Continuous prediction gives the order of cells continuously distributed within each phase (Sakaue-Sawano et al., 2008). The order could be used to locate single cells along the circular cell cycle trajectory, which we called pseudo time in the cell cycle. Continuous assignment methods includes cyclum (Liang et al., 2020), cyclops (Anafi et al., 2017), peco (Hsiao et al., 2020), and Oscope (Leng et al., 2015). Cyclum and cyclops use an unsupervised learning technique autoencoder to analyze the cell-gene expression matrix. To identify cell cycle phases in the scRNA-seq data, Cyclum projects high-resolution single cells onto a low-dimensional cyclic periodic trajectory, where the pseudo times are represented by radians in the range [0, 2π] (Liang et al., 2020). Unlike cyclum, cyclops uses linear projection to project data onto a closed elliptical curve in low-dimensional space (Anafi et al., 2017). It employs square root and division in the autoencoder model, which makes optimization more complex. Peco is a supervised approach that uses the data generated from FUCCI fluorescence images and scRNA-seq to train the “naive Bayes” predictor for predicting the continuous cell cycle phase (Hsiao et al., 2020). The supervised approach can ensure the accuracy of cell cycle prediction, while cannot be applied to scRNA-seq data without knowing cell cycle label. Oscope identifies oscillating genes and uses them to order single cells at different cell cycle phases (Leng et al., 2015). Each pair of genes needs to be tested for compliance with the circular patterns, resulting in computational complexity. Beyond the continuously quantitative description of cell cycle progress, classification of cells into discrete states is also applied in the study of cell cycle identification. Cyclone classifies single cells into different cell cycle stages according to the selected marker gene pairs (Scialdone et al., 2015). As more and more cell cycle marker genes are discovered, the library of cell cycle marker genes can be expanded and updated. Thus, cyclone can be applied to cell cycle estimation of more species and the prediction accuracy of cell cycle expects to be improved. Liu et al. introduced cell cycle time-series as a consensus traveling salesman problem (TSP) to recover cell cycle pseudo time (reCAT) from single-cell transcriptome data. In their study, a hidden Markov model (HMM) based on Bayes-scores and mean-scores was designed to segment the pseudo times into G1, S, and G2/M (Liu et al., 2017). Due to the complexity of the reCAT model, there are many parameters that need to be set manually in advance, which brings a great challenge to the accuracy of the model.

### Strategy Development to Remove Cell Cycle Effects From scRNA-seq Data

Single-cell RNA sequencing made it possible to study heterogeneity in gene expression in high resolution. Such heterogeneity exists due to technical noise and different biological factors (Buettner et al., 2017). The cell cycle is a major source of bias, which introduces greater within-cell-type heterogeneity, causing quite different expression profiles between cell types (Barron and Li, 2016). For example, in the study of the differentiation of *T*_*H*_ cells, the cell cycle is a factor affecting cell heterogeneity. If the cell cycle effect is not considered to remove, genes associated with cell cycle can lead to bias in cell clustering, thus affecting the accuracy of cell differentiation studies (Barron and Li, 2016). This problem also exists in studies of cell type identification, tumor development, etc. Three major methods have been developed to remove the cell cycle effect based on gene expression profiles, including Seurat (Butler et al., 2018), ccRemover (Barron and Li, 2016), and Cyclum (Liang et al., 2020). Seurat and ccRemover are the most popular ones. Both of them rely on the known cell-cycle genes. Seurat calculated cell cycle phase scores based on S and G2M cell cycle markers. Cells that do not express these markers may be in the G1 phase. Cell cycle effects are removed during data normalization (Butler et al., 2018). ccRemover is a PCA-based method that identifies components related to the cell cycle with a larger component score by comparing with the control group. Thus, it can retain other factors while thoroughly removing the cell cycle effect (Barron and Li, 2016). Cyclum removes the cell cycle effect by subtracting the reconstructed matrix of non-linear components in the hidden layer from the expression level. Application to virtual tumor data shows that cyclum can more accurately eliminate cell cycle effects on cell clusters than Seurat and ccRemover. It can accurately distinguish two subclones in the virtual tumor data (Liang et al., 2020).

Considering various aspects, Cyclum is a competent method both in predicting cell cycle phases and removing cell cycle factors in cell clustering analysis. Also, this is an unsupervised method that can be used in cell cycle estimation of single-cell data without label information, and it does not suffer from computational complexity and the need to set multiple parameters manually.

## Cell Type Identification

Identifying cell types from single-cell transcriptomic data is a basic goal of scRNA-seq data analysis. Classifying cells is crucial to learn more about tissue functions and to reveal mechanisms underlying pathological states. Knowledge of known cell types allows us to cluster biomarkers for specific cell types, and provides insight into novel cell discovery and obtain cellular profiles of cell lineages, organs, and even whole tissue (Cao et al., 2017, 2019; Fincher et al., 2018; Han et al., 2018; Plass et al., 2018; Tabula Muris Consortium et al., 2018). However, manual annotation of cell types is so cumbersome and time-consuming. Therefore, the numbers of computational classification methods are rapidly growing to identify cell types of scRNA-seq data. Unsupervised algorithms are typically used to cluster cells into different clusters based on the similarity between cells, followed by cell type identification by assigning discrete cell-type labels to each cluster. So far, more than 20 methods have been proposed to identify cell types from scRNA-seq data (Abdelaal et al., 2019). Clustering algorithms such as k-means and DBSCAN (Ester et al., 1996) are commonly used to group cells into different cell types. RaceID (Grun et al., 2015) is a typical example of applying k-means clustering algorithm to give the cell-type annotation for individual cells. SNN-Cliq (Xu and Su, 2015) takes the effect of nearest neighbors into account and cluster cells on the high-dimensional scRNA-seq data. SINCERA (Guo et al., 2015) performs a hierarchical clustering on the similarity matrix computed by Pearson correlation. However, such algorithms may require non-intuitive parameters, such as the number of clusters and the initial centroids.

The high resolution of scRNA-seq data is another challenge in the identification of cell types. To solve this challenge, several methods have taken dimension reduction into account. Single-cell consensus clustering (SC3) (Kiselev et al., 2017) and Seurat (Butler et al., 2018) are applied to identify cell types by using different combinations of the clustering algorithm, dimensionality reduction, and feature selection. SC3 combines k-means and spectral clustering algorithms to identify subclones from the transcriptomic data of neoplastic cells. Seurat (Butler et al., 2018) utilizes t-distributed stochastic neighbor embedding (t-SNE) technology (Maaten and Hinton, 2008) for dimension reduction and DBSCAN (Ester et al., 1996) is applied to cluster cells in the reduced dimension. In addition to identifying cell types, Seurat has evolved into a versatile single-cell analysis tool that facilitates users in data pre-processing, cell cycle removal, differential gene analysis, etc.

Neural networks have also proven to be effective in identifying cell types from scRNA-seq. Ma and Pellegrini (2020) proposed ACTINN for automated identification of cell types from scRNA-seq data. ACTINN employs a neural network with three hidden layers and is trained by a set of scRNA-seq data with predefined cell types. The trained parameters make it convenient for ACTINN to be applied to other datasets. Lopez et al. (2018) proposed scVI, a hierarchical Bayesian model based on deep neural networks, for the probabilistic representation and analysis of gene expression in single cells. It also considers both library size and batch effect, which are two major issues in scRNA-seq data analysis.

In short, when analyzing cell types from a single cell dataset, if the number of cell types is known, the easiest and fastest way is to directly use clustering algorithms, such as k-means and DBSCAN. Otherwise, a dimensionality reduction algorithm is required. If several analyses on the same single-cell data are needed besides cell-type identification, Seurat software is highly recommended. When studying cell types in more detail, such as subtype analysis, ADMM appears to be more accurate than other traditional unsupervised algorithms in predicting cell types and subtypes.

## Other Applications of Machine Learning in Downstream Analyses of Single-Cell Sequencing Data

### Copy Number Variance Estimation and Subclone Analysis

Copy number variance (CNV) is a major class of genetic drivers of cancer, so it is very important in cancer research. Single-cell DNA sequencing technologies allow the detection of genomic variants such as CNVs. Several methods have been developed for CNV detection from single-cell DNA data, such as Ginkgo (Garvin et al., 2015), HMMcopy (Lai and Ha, 2013), CopyNumber (Nilsen et al., 2012), and SCOPE (Wang R. et al., 2020). Ginkgo can automatically construct copy-number profiles and phylogenetic trees of related cells from scDNA-seq data. One of the advantages of HMMcopy is its ability to infer both segmentation and absolute copy numbers (Lai and Ha, 2013). SCOPE is another method for copy-number estimation from scDNA-seq data, which has successfully reconstructed cancer subclones. Since there are technological challenges in performing simultaneous scRNA-Seq and scDNA-Seq analysis experimentally from a single cell, several methods have been proposed to detect genomic heterogeneity from scRNA-Seq data. However, identification of CNVs from RNA-Seq data is very challenging because it is difficult to capture deletion/amplification and dynamic changes in gene expression in RNA-Seq data, even more difficult for scRNA-seq data. Fan et al. (2018) proposed a computational approach called HoneyBADGER that implements an allele-based HMM and a hierarchical Bayesian model to identify copy number variation and loss of genomic heterozygosity of single cells from scRNA-seq data. We recently developed CaSpER (Serin Harmanci et al., 2020) for single-cell CNV inference from single-cell RNA sequencing data. CaSpER applies a novel and efficient method to generate allele shift signal profile, which quantifies genome-wide heterozygosity loss (Serin Harmanci et al., 2020). The outstanding contribution of CaSpER is that it does not require heterozygous variant calling to generate allelic shift profiles (Serin Harmanci et al., 2020). CaSpER is a highly recommended method since it can be used to not only identify gene expression signatures of mutually exclusive CNV sub-clones, but also analyze gene ontology enrichment.

### Phylogenetic Tree Inference

The rapid development of single-cell genomic and transcriptomic technologies has paved the way for the emergence of studying the evolutionary process of cells in cancer or an organism, which is known as cell phylogenetic tree or lineage tree inference. There is a strong need to infer the tree structure of cell lineages with single-cell sequencing data to classify evolutionary trajectory of the organisms or cancer progression. With the growth of research fervor in cell lineage tree inference, several machine learning methods have been proposed. Most of the existing methods such as SCITE (Jahn et al., 2016) and SiFit (Zafar et al., 2017) are designed on the basis of Markov chain Monte Carlo (MCMC) approach. SCITE and SiFit have the disadvantage that they cannot handle massive single-cell data and their ideas are based on the assumption of uniformly distributed errors in genotypes. However, genotypes derived from single-cell sequencing data tend to have non-uniform uncertainty (Singer et al., 2018). Monovar quantifies the genotype likelihood values for each cell based on the assumption that sequence data at different sites are completely independent (Wu, 2020). ScisTree, a newly developed method, adopts the statistical learning approach to find the maximum likelihood to infer cell phylogenetic tree and call genotypes from noisy single-cell genotype data with its own individualized probability (Wu, 2020). This allows ScisTree to deal with uncertain genotypes, where the content of single-cell sequencing data may vary at different cells and sites.

Sadeqi Azer et al. (2020) inferred the most likely tumor phylogeny via deep learning and eliminate noises such as dropout events in alleles and low sequence coverage issues with a maximum likelihood/parsimony approach. The noise reduction processes target the possible set of false negative/false-positive variant calls to ensure constructing a reliable phylogenetic tree. Satas et al. (2020) developed an algorithm called SCARLET to infer tumor phylogenies from single-cell DNA sequencing data while taking into account both CNA-driven loss of SNV and sequencing errors. Campbell et al. (2019) developed a statistical learning tool called Clonealign that uses single-cell RNA and DNA sequencing data to assign gene expression states to cancer clones. Simultaneously applying DNA and RNA sequencing data to infer the phylogenetic tree is still a challenging issue. Novel computational tools are needed to map parallel single-cell DNA and RNA sequencing data from independent cell populations for exploring genome-transcriptome association.

### Lineage Trajectory Inference

Inferring the position of each individual cell on the lineage trajectory based on the scRNA-seq profiles is one of the promising applications of scRNA-seq. Dynamic processes such as cell cycle, cell differentiation, and cell activation (Etzrodt et al., 2014; Tanay and Regev, 2017) can be modeled computationally using trajectory inference methods. The inferred trajectories can be cyclic, linear, bifurcating, tree-structured or disconnected graphs.

Numbers of trajectory inference methods have come out over the past few years. Most of them focus on estimating the trajectory and correctly ordering the cells along the trajectory. The well-known tool Monocle 2 (Qiu et al., 2017)developed by Trapnell et al. uses DDRTree (Mao et al., 2015), a scalable reversed graph embedding algorithm, for finding the projections between gene expression profiles and lower-dimensional spaces and learning a principal tree on a population of single cells in this reduced space. Chen et al. proposed a new method named Landmark Isomap for Single-cell Analysis (LISA). The performance of LISA is more applicable to large single-cell datasets. LISA applied Isomap to construct the low feature dimension and built Minimum spanning tree (MST) on the cluster centers similar to monocle2. Comparing to monocle2, LISA is faster and uses less memory (Chen Y. et al., 2019). Single-cell Trajectories Reconstruction, Exploration and Mapping (STREAM) is an interactive pipeline capable of reconstruct complex branch trajectories from single-cell transcriptomics and epigenome data, providing a new concept of transition genes, whose expressions across cells have a high correlation with the predicted pseudotime (Chen H. et al., 2019). New cells can be mapped to the STREAM-inferred trajectories without reconstruction.

Several other methods have also been proposed to infer topology of scRNA-seq data (Street et al., 2018; Cao et al., 2019; Wolf et al., 2019). These methods identify the order of cells along branches and obtain topological connection between these branches. Partitioned approximate graph abstraction (PAGA) was proposed to construct a KNN graph on cells and then perform Louvain clustering algorithm to identify the membership of cells. Both continuous and disconnected structures are preserved in data at multiple resolutions (Wolf et al., 2019). One of the major updates in Monocle3 (Cao et al., 2019) than Monocle2 is the use of PAGA to automatically partition cells to learn disjoint or parallel trajectories. As a result, Monocle3 can reconstruct trajectories for complex and massive single-cell datasets. Monocle 3 was applied to depict mouse organogenesis cell atlas using ∼2 million cells generated from 61 embryos staged in E9.5–E13.5 and established a global trajectory from E9.5 to E13.5 and subtrajectories for all major developmental systems. RNA velocity (La Manno et al., 2018) is a novel concept and has been developed to infer cell RNA dynamics based on the deviation of the observed ratio of spliced and unspliced mRNA from an inferred steady state. A recently introduced method, scVelo, breaks the central assumptions of a common splicing rate in RNA velocity. scVelo generalizes RNA velocity to transient cell states through a likelihood-based dynamical model (Bergen et al., 2020).

In general, there is no single method that predicts all structures of the trajectory, and no single method that works for all the datasets. Two important factors need to be considered when inferring the cell trajectories. One is the structure of the trajectory. PAGA can be used to predict the most cell trajectory types (Saelens et al., 2019). The other one is the sample size of the dataset. Monocle3 is recommended to infer complex trajectories and sub-trajectories for massive scRNA-seq data. While RNA velocity and scVelo are designed specifically for spliced and unspliced mRNA matrices.

### Cell–Cell Interaction

Cell–cell interaction (CCI), also known as cell–cell communication, governs the functional activities of cells and coordinates multiple-cell actions (Singer, 1992). The dynamic network constructed through interaction and connections between cells with adjacent or remote partners is significant important in numerous biological activities (Camp et al., 2017). Gene expression data in many tissues and organs, such as the brain (Yuzwa et al., 2016; Sheikh et al., 2019), heart (Skelly et al., 2018), and lungs (Zepp et al., 2017; Cohen et al., 2018), has revealed that CCI plays an important role in organ function. Studying CCI within disease/tumors and their surrounding microenvironments can bring to light how cells communicate with their surroundings and help guide the development of effective treatment strategies (Kumar et al., 2018). CCI leverages diverse molecules, including ions fluxes, metabolites, secreted vesicles, etc. A majority part of the interaction is mediated by secreted ligands and receptors (Armingol et al., 2020). The fast development of scRNA sequencing technologies provides an unparalleled opportunity to infer the ligand–receptor (LR) interactions at a high-resolution cell state map.

Many statistics tools have been developed to perform such inferences (Wang Y. et al., 2019; Cabello-Aguilar et al., 2020; Efremova et al., 2020; Hou et al., 2020; Jin et al., 2020). iTALK (Wang Y. et al., 2019) adopts a product score to deduce ligand-receptor (LR) pairs within the highly expressed genes using public LR databases. The input of iTALK is a count expression matrix with known cell type information. It has the ability to process multiple datasets, and can handle with batch effects and variability in LR expression. CellPhoneDB (Efremova et al., 2020) predicts LR interactions between cell types according to the degree of their expression, taking subunit architecture of the ligands and receptors into consideration at the same time. The author generated null distribution for each LR pair between cell types, and obtained the probability of cell-type pattern of each LR to predict enriched signaling interactions. NATMI (Hou et al., 2020) also uses the transcriptome profiling of each LR in scRNA-seq dataset with labeled cell types, and then predicts the connections from a ligand sending cell to a receptor cell among all cell types, and finally generates a cell connectivity summary network matrix. SingleSignalR (Cabello-Aguilar et al., 2020) is an R package designed to infer and visualize LR interaction. This tool first integrates the existing LR pairs databases as LRdb, and computes the mean expression of marker genes among all the cells to obtain a regularized score for each LR pair with additional cell-types specific information. CellChat, an R package, was developed to infer, visualize and analyze CCI for scRNA-seq data (Jin et al., 2020). It also provides a more extensive database, which contains multi-subunit structure of LR complex and stimulatory and inhibitory cofactors. CellChat applied a mass action-based model to infer the probability of cell-state related signaling interactions between LR pairs.

Each method has its own advantage over others. iTALK can be applied to time series data and different platform data, but it does not propose any cutoff for the scores of the LR interactions. CellPhoneDB provides online analysis and considers the heteromeric composition of the ligands and receptors. NATMI is a network-based tool used to estimate which cell type pairs are most likely to communicate. The regularized score of SingleSignalR has better performance on control false positives over other tools. Beyond the purely fundamental research enterprise of interpreting the cell–cell biological messages, CellChat can be used to compare communication networks in different cell-states of an organ.

### Gene Regulatory Network

Studies have revealed that genes cannot work alone (Cordell, 2009). Instead, they constantly influence one another, which can be called epistasis (Phillips, 2008), involving the interaction between two or more genes. These interplays are important for molecular regulation, signal transduction, biological networks, and lots of functional pathways (Harvey et al., 2013). Therefore, network modeling of genes is significantly helpful for our understanding of key regulators related to biochemical pathways. Gene regulatory network (GRN) (Fiers et al., 2018) describes a set of interacting regulatory genes with specific cellular function. GRN has been extensively utilized based on graph model for functional analysis in recent years. GRN is essential to revealing questions of cellular identity (Han et al., 2020) and has been demonstrated to play important roles in searching for disease-related biomarkers and drug position targets (Cha and Lee, 2020) and extensively utilized as an important tool for analyzing genomics data. Network modeling has long been employed as a powerful tool to understand and interpret complex biological systems (Huynh-Thu et al., 2010; Lim et al., 2016; Matsumoto et al., 2017; Woodhouse et al., 2018; Moerman et al., 2019; Mohammadi et al., 2019). Boolean models, including Single-Cell Network Synthesis (SCNS) toolkit (Woodhouse et al., 2018) and BoolTraineR (Lim et al., 2016), focus on discovering potential combinations of transcription factors (TFs) which could be taken as connected nodes in the network. SCODE (Matsumoto et al., 2017) is a regulatory network inference algorithm and relies on an ordinary differential equation (ODE) model to predict regulatory networks using differentiating cells in scRNA-seq data. This method provides a command-line interface which can facilitate analysis. Regression-based network modeling algorithms, such as GENIE3 and GRNBoost2 assume that gene expression can be represented as a linear combination. GENIE3 (Huynh-Thu et al., 2010) was designed for bulk RNA-seq data analysis and also applied to scRNA-seq data. GENIE3 uses Random Forests algorithm to construct a regulatory network by decomposing selected genes into the same number of regression problems. In each regression problem, one gene is used as a response variable to be predicted based on all the other genes. GRNBoost2 (Moerman et al., 2019) is a self-tuning algorithm which uses gradient boosting instead of estimating the decision trees from a global perspective based on GENIE3 architecture. SCINET (Mohammadi et al., 2019) implements co-expression and motif enrichment analysis to directly predict the interactions between TFs and their targeting genes. SCENIC (Aibar et al., 2017) is a correlation-based method which combines gene co-expression with TF-binding motif analyses to identify GRN modules and predict TF regulators from scRNA-seq data. SINGE is a computational tool which adopts kernel-based Granger Causality regression to smooth irregular pseudotimes and dropout values to reconstruct gene regulatory network (Deshpande et al., 2021). SINGE has a better performance over other GRN inference methods in evaluating ChIP-seq and ChIP-chip data.

## Integrated Analysis of ScRNA-Seq and Spatial Transcriptome

Considerable technological advances in sequencing technologies have made it possible for researchers to study the transcriptomic landscape at spatial resolution recently (Lubeck et al., 2014; Chen et al., 2015; Patrik et al., 2016; Svensson et al., 2018; Eng et al., 2019; Sun et al., 2020). Spatial transcriptomics (ST) technologies attracted lots of attention in the year 2020 (No authors listed, 2021) and have changed the way we understand the architecture of complex tissues. ST technologies have the potential to describe cellular organization and functioning in intact multicellular environments and elucidate interactions between gene expression and cellular environment. Several methods have been proposed to integrate scRNA-seq with spatial transcriptomics to study the heterogeneity of intact tissue (Asp et al., 2019; Cable et al., 2020; Hunter et al., 2020; Ji et al., 2020; Moncada et al., 2020; Su and Song, 2020). The common way is to estimate reference cell type/cluster signatures from scRNA-seq profile, and then map the signatures onto spots to decompose ST at single-cell resolution. By adding spatial information to scRNA-seq data, spatial transcriptomics has transformed our understanding of tissue functional organization and CCI *in situ*. Ji et al. (2020) identified a heterogeneous tumor leading edge composed of tumor-specific keratinocyte (TSK) and basal tumor cells and a TSK-proximal fibrovascular niche using spatial transcriptomics data. Coincidentally, Hunter et al. (2020) integrated scRNA-seq data with ST to construct an atlas architecture within the tumor and their neighboring surrounding, and identified a unique transcriptomics interface region. These findings have the potential to disclosure the mechanism of tumor invasion and development. Due to the limitation in terms of sequence coverage and overall throughput, it is difficult to get the true single-cell resolution for the whole intact tissue sample, such as exactly numbers of cells and cell types from each spot. However, with the increasing development of technology, we believe these will not big problems. Coupling single-cell sequencing approaches with ST has enormous potential to improve current modeling at single-cell resolution, such as CCI and GRN analysis.

## New Challenges of Further Single Cell Data Analysis

The machine intelligence for single cell sequencing data analysis is still growing at a fast pace. We still face more challenges in processing and analyzing such data. Here we summarize several aspects of the challenges.

### Data Imputation

Although there have been many imputation algorithms for single-cell expression data, imputing single cell data at reads level still lacks. It’s challenging to determine the true abundance of the transcripts even if the transcript structures are known (Roberts et al., 2011). The extremely low reads coverage with the much higher bias of scRNA-seq experiments makes transcript quantification more challenging. Specifically, if the missing reads are not imputed, metrics of transcript quantification lead to underestimation of gene expressions. Therefore, accurate transcript quantification requires recovery of the missing reads throughout the gene to assure the even and compatible coverages within and across the exons and transcripts.

### Single-Cell Multi-Omics Integration

The purpose of the integrated analysis is to solve important biological problems using proper methods. Therefore, the inherent biological differences related to different tissues, species, and molecular layers (such as RNA-seq and ATAC-seq) need to be considered.

### Trajectory Inference

There are several future challenges in trajectory inference need to be taken into account. First, compared with the actual cell number of transcriptome analysis, many existing methods only allow the measurement of a very limited number of cells. Second, it is necessary to define the features to be used in constructing trajectories. Features with the same expression patterns usually retain important information of cells that belong to the same lineage. Third, there should be a definite evaluation method to compare the performance of different trajectory inference algorithms as previously described by Saelens et al. (2019).

In addition, technical noise and data parallel processing are problems faced by all single-cell data analysis. Technical noise can affect the accuracy of downstream analysis of single-cell data. Since single-cell data usually contains more than thousands of features, it is important to speed up the single analysis with parallel processing. In summary, we reviewed the application of machine learning methods and tools in single-cell sequencing data imputation and downstream data analysis, as well as existing potential challenges. Spatial distribution and building structure are very important for understanding the development of human diseases. Therefore, single-cell data analysis of spatial transcriptomics will become the next wave of computational tool development.

## Author Contributions

JL, ZF, and XZ are the main writers of the review, complete the collection and analysis of relevant literatures, and write the first draft of the manuscript. WZ helped in writing and revising the manuscript. All authors discussed the revision and contributed to the final manuscript.

## Conflict of Interest

The authors declare that the research was conducted in the absence of any commercial or financial relationships that could be construed as a potential conflict of interest.
